# Modulation of Cardiovascular Function in Primary Hypertension in Rat by SKA-31, an Activator of *K_Ca_2.x* and *K_Ca_3.1* Channels

**DOI:** 10.3390/ijms20174118

**Published:** 2019-08-23

**Authors:** Monika Kloza, Marta Baranowska-Kuczko, Marek Toczek, Magdalena Kusaczuk, Olga Sadowska, Irena Kasacka, Hanna Kozłowska

**Affiliations:** 1 Department of Experimental Physiology and Pathophysiology, Medical University of Białystok, 15-222 Białystok, Poland; 2 Department of Clinical Pharmacy, Medical University of Białystok, 15-222 Białystok, Poland; 3 Department of Pharmaceutical Biochemistry, Medical University of Białystok, 15-222 Białystok, Poland; 4 Department of Histology and Cytophysiology, Medical University of Białystok, 15-222 Białystok, Poland

**Keywords:** SHR, hypertension, SKA-31, *K_Ca_3.1*/*K_Ca_2.3*-EDH–dilator system, K_IR_, Na^+^/K^+^-ATP-ase

## Abstract

The aim of this study was to investigate the hemodynamic effects of SKA-31, an activator of the small (*K_Ca_2.x*) and intermediate (*K_Ca_3.1*) conductance calcium-activated potassium channels, and to evaluate its influence on endothelium-derived hyperpolarization (EDH)-*K_Ca_2.3*/*K_Ca_3.1* type relaxation in isolated endothelium-intact small mesenteric arteries (sMAs) from spontaneously hypertensive rats (SHRs). Functional in vivo and in vitro experiments were performed on SHRs or their normotensive controls, Wistar-Kyoto rats (WKY). SKA-31 (1, 3 and 10 mg/kg) caused a brief decrease in blood pressure and bradycardia in both SHR and WKY rats. In phenylephrine-pre-constricted sMAs of SHRs, SKA-31 (0.01–10 µM)-mediated relaxation was reduced and SKA-31 potentiated acetylcholine-evoked endothelium-dependent relaxation. Endothelium denudation and inhibition of nitric oxide synthase (eNOS) and cyclooxygenase (COX) by the respective inhibitors *l*-NAME or indomethacin, attenuated SKA-31-mediated vasorelaxation. The inhibition of *K_Ca_3.1*, *K_Ca_2.3*, K_IR_ and Na^+^/K^+^-ATPase by TRAM-34, UCL1684, Ba^2+^ and ouabain, respectively, reduced the potency and efficacy of the EDH-response evoked by SKA-31. The mRNA expression of eNOS, prostacyclin synthase, *K_Ca_2.3*, *K_Ca_3.1* and K_IR_ were decreased, while Na^+^/K^+^-ATPase expression was increased. Collectively, SKA-31 promoted hypotension and vasodilatation, potentiated agonist-stimulated vasodilation, and maintained *K_Ca_2.3*/*K_Ca_3.1*-EDH-response in sMAs of SHR with downstream signaling that involved K_IR_ and Na^+^/K^+^-ATPase channels. In view of the importance of the dysfunction of endothelium-mediated vasodilatation in the mechanism of hypertension, application of activators of *K_Ca_2.3*/*K_Ca_3.1* channels such as SKA-31 seem to be a promising avenue in pharmacotherapy of hypertension.

## 1. Introduction

The healthy endothelium regulates vascular tone by the generation of vasodilation factors like nitric oxide (NO), prostacyclin (PGI_2_) and/or endothelium-dependent hyperpolarization (EDH), among others [1]. EDH-type relaxation occurs in small resistance arteries and is thus an important modulator of systemic blood pressure (BP) and blood flow. EDH functions independently of NO and prostanoids, but it involves endothelial small (*K_Ca_2.3*) and intermediate (*K_Ca_3.1*) conductance calcium-activated potassium channels (K_Ca_). K_Ca_ activation and accompanying endothelial hyperpolarization can spread to the surrounding smooth muscle via myoendothelial gap junctions activating inward-rectifying potassium ion channels (K_IR_2.1) and/or a Na^+^/K^+^-ATPases, thereby inducing relaxation [2]. Moreover, it has long been known that, acting mainly via *K_Ca_3.1*, EDH is a primary mediator of myoendothelial feedback in resistance arteries to limit agonist-induced depolarization and constriction [3]. 

Endothelial dysfunction plays an important role in the pathogenesis of hypertension [4]. Hypertension is a major cause of the increasing morbidity of cardiovascular diseases in modern societies and if left untreated, leads to premature death [5]. It is believed that in human hypertension as well as in the spontaneously hypertensive rat (SHR), which is the most widely studied animal model of hypertension, endothelium-dependent relaxation may be attenuated. One of the main causes for endothelial dysfunction is the reduced availability of NO [6] and/or defects in the *K_Ca_3.1*/*K_Ca_2.3*-EDH–dilator system including altered expression i.e., of *K_Ca_2.3* and *K_Ca_3.1* [1]. Mice lacking *K_Ca_3.1*-and/or *K_Ca_2.3* are reported to manifest with severe impairment of the EDH-dilatory responses and increased mean arterial blood pressure [7,8]. Functional evidence for targeting pathway of impaired *K_Ca_3.1*/*K_Ca_2.3*-EDH–dilator system would raise the possibility to improve vascular function [3] and could serve the way for new treatment for hypertension and related cardiovascular diseases [9]. 

Pharmacological activation of the *K_Ca_3.1* and *K_Ca_2.3* channels could improve endothelium dysfunction and BP regulation thereby representing novel targets for antihypertensive drugs [10,11,12]. The *K_Ca_3.1* and *K_Ca_2.x* channel activator SKA-31 (naphtho (1,2-d)thiazol-2-ylamine) exhibits excellent pharmacokinetic properties such as long half-life (12 h), no toxicity, and low plasma protein binding in rodents [13]. Moreover, it effectively reduces blood pressure in normotensive mice, dogs, and pigs [13,14,15] and in mice with hypertension induced by angiotensin II [13], connexin40 deficiency [16]. SKA-31 is also shown to produce a transient decrease in mean arterial BP that was accompanied by either a reflex tachycardia [14], bradycardia [16] or unchanged heart rate [13,15,17]. Using arterial pressure myography, it has been shown that SKA-31 increased coronary flow in a *K_Ca_3.1*-dependent manner in diabetic rats [18], evoked concentration-dependent inhibition of myogenic tone in the rat cremaster and middle cerebral arteries [19] and in small mesenteric arteries (sMAs) in a *K_Ca_2.3*/*K_Ca_3.1*-dependent manner [20]. SKA-31 potentiated endothelium-derived hyperpolarization (EDH-type dilatory response) elicited by acetylcholine (Ach) in dog mesenteric and mouse carotid artery [13,14], respectively.

In view of the importance of the dysfunction of endothelium-mediated vasodilatation, including EDH-response in hypertension, application of activators of *K_Ca_2.3*/*K_Ca_3.1* channels such as SKA-31 seem to be promising avenue in pharmacotherapy of hypertension. In this respect, the principal aim of our study was to investigate the influence of primary hypertension on SKA-31-mediated systemic hemodynamic effects in anesthetized rats, and also to investigate putative endothelium-dependent mechanisms, including EDH-*K_Ca_2.3*/*K_Ca_3.1* type relaxation in isolated endothelium-intact small mesenteric arteries (sMAs).

## 2. Results

### 2.1. General

**Figure 1:**
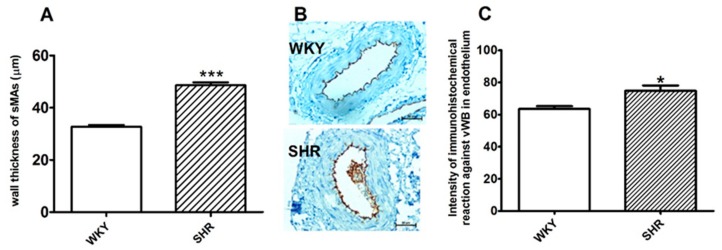
Measurement of medial width (**A**), representative micrographs (**B**) and intensity of the immunohistochemical reaction for the von Willebrand factor (vWF) (**C**) in the small mesenteric arteries (sMAs) from normotensive Wistar Kyoto rats (WKY) and spontaneously hypertensive (SHR) rats. Mean ± SEM of *n* = 4–5 animals for each bar (**A,C**); * *p* < 0.05, *** *p* < 0.001, compared to the WKY; bar = 50 μm.

The arterial systolic BP of the SHR measured by tail cuff method was higher than the age-matched WKY rats (approximately 189 ± 7 mmHg; *n* = 30 vs. 104 ± 5 mmHg; *n* = 31, respectively. The hypertension increased medial hypertrophy in sMAs by approximately 15% compared to the normotensive control (Figure 1A). Representative images of the vascular remodeling and vWF immunoreactivity of sMAs are shown in Figure 1B. The intensity of vWF-related immunoreactivity was higher by approximately 18% in endothelial cells of sMAs from SHR relative to normotensive controls (Figure 1C).

### 2.2. Influence of SKA-31 on BP and HR of SHR and WKY Rats

**Figure 2:**
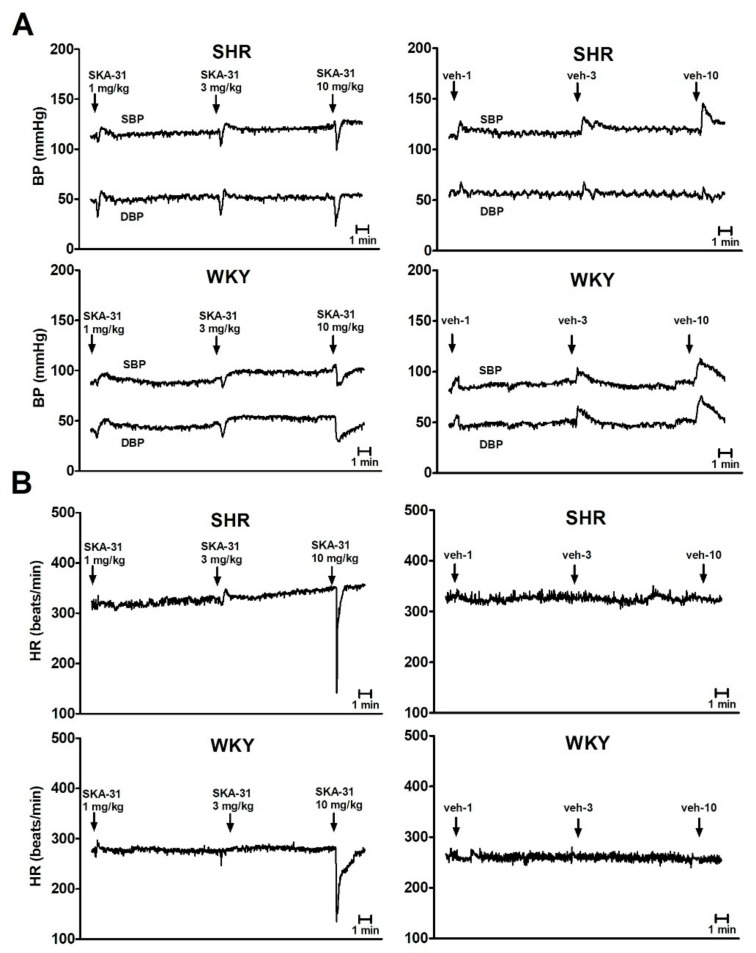
Traces from representative experiments showing the influence of SKA-31 (1, 3, or 10 mg/kg i.v.) or its vehicle (veh-1, veh-3, or veh-10, respectively) on (**A**) diastolic blood pressure (DBP) and systolic blood pressure (SBP) or (**B**) heart rate (HR) in urethane-anaesthetized spontaneously hypertensive rats (SHR) and Wistar Kyoto rats (WKY). Arrows show the moment of injection of the of SKA-31/veh.

**Figure 3:**
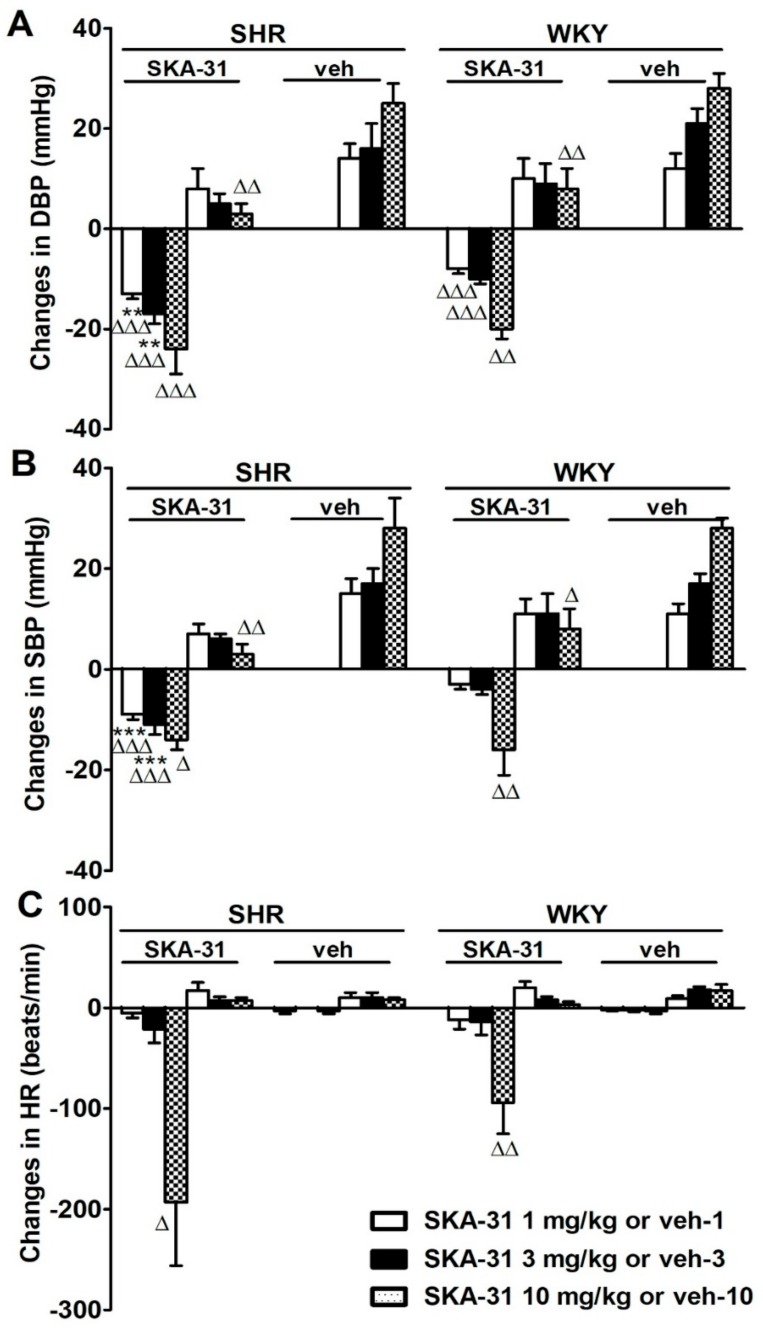
Influence of SKA-31 (1, 3, 10 mg/kg; i.v.) or vehicle on (**A**) diastolic blood pressure (DBP), (**B**) systolic blood pressure (SBP) and (**C**) heart rate (HR) of urethane-anaesthetized spontaneously hypertensive rats (SHR) and Wistar Kyoto rats (WKY). Mean ± SEM, *n* = 16, ** *p* < 0.01, *** *p* < 0.001 compared to respective WKY group; ^Δ^ *p* < 0.05, ^ΔΔ^ *p* < 0.01, ^ΔΔΔ^ *p* < 0.001 compared to respective group receiving vehicle for SKA-31.

Under urethane anesthesia, basal systolic BP, diastolic BP, mean BP and HR were higher in SHR compared to WKY rats. These parameters were stable throughout the whole experiment (Table 1). Injections of the appropriate volume of vehicle matched for each dose of SKA-31 increased both DBP and SBP comparably in both groups. On the contrary, administration of SKA-31 (1, 3 and 10 mg/kg) caused initially a brief, dose-dependent decrease in DBP and SBP (Figure 2A and Figure 3A,B). This decrease was higher in SHR than in WKY rats for 1 and 3 mg/kg of SKA-31. The subsequent increase in BP induced by SKA-31 injections was lower than that evoked by vehicle (Figure 3A,B). Only the highest dose of SKA-31 (10 mg/kg) evoked a profound and short-term decrease in HR amounting to about 50% and 40% of basal values in SHR and WKY rats, respectively (Figure 2B and Figure 3C).

### 2.3. Influence of Endothelial Physical Disruption, INDO and l-NAME on SKA-31-Induced Relaxation

**Figure 4:**
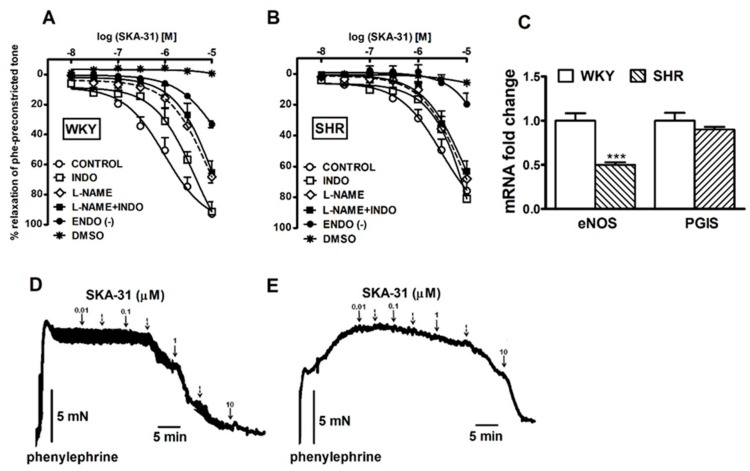
Influence of indomethacin (INDO, inhibitor of cyclooxygenase, COX, 10 μM) and *N*^ω^-nitro-*L*-arginine methyl ester (*l*-NAME, inhibitor of nitric oxide synthase, NOS, 100 μM) given alone or together, or endothelium physical disruption on the vasorelaxant effects of SKA-31 in the small mesenteric arteries (sMAs) from WKY (**A**) and spontaneously hypertensive (SHR) rats (**B**). RT-qPCR analysis of *eNOS* and *PGIS* in vessels isolated from SHR and WKY rats. Results shown as relative fold change in mRNA expression in SHR in comparison to WKY controls, where expression level was set as 1.0 (**C**). The representative original traces of the SKA-31 induced relaxation of sMAs derived from WKY (**D**) and SHR (**E**). Arrows show the moment of application of the particular concentrations of SKA-31. Broken arrows represent the following concentrations (from the left): 0.03, 0.3, 3 µM, respectively. Results are expressed as a percentage of relaxation of the isometric contraction induced by phenylephrine (Phe). Mean ± SEM of *n* = 5–14 animals for each curve or mRNA levels. In a few cases, the SEM is smaller than or equal to the size of symbols. *** *p* < 0.001 compared to WKY. See Table 2 for statistical analyses.

SKA-31 (0.01–10 µM) induced a robust, concentration-dependent nearly full relaxation in endothelium-intact isolated sMAs preconstricted with phenylephrine in normotensive WKY (92% relaxation) and hypertensive SHR rats (75% relaxation). SKA-31 had significantly greater vasorelaxation potency in WKY compared to SHR rats (*p* < 0.001). Endothelial denudation attenuated the SKA-31-induced maximal relaxation in sMAs by approximately 65% and 75% in WKY and SHR rats, respectively. The concentration-response curves (CRCs) were unaltered in the solvent treated (DMSO) condition (Figure 4A,B for the pEC_25_ and R_max_ see values in Table 2; the original traces of the SKA-31-induced relaxation see in Figure 4D,E).

Indomethacin (INDO, 10 µM) shifted CRCs for SKA-31 to the right by a factor of 5 in WKY and 2.5 in SHR rats, decreasing potency in comparison to the corresponding control group. The maximal relaxation was not modified in sMAs in the both groups (Figure 4A,B, for the pEC_25_ and R_max_ see values in Table 2).

**Figure 5:**
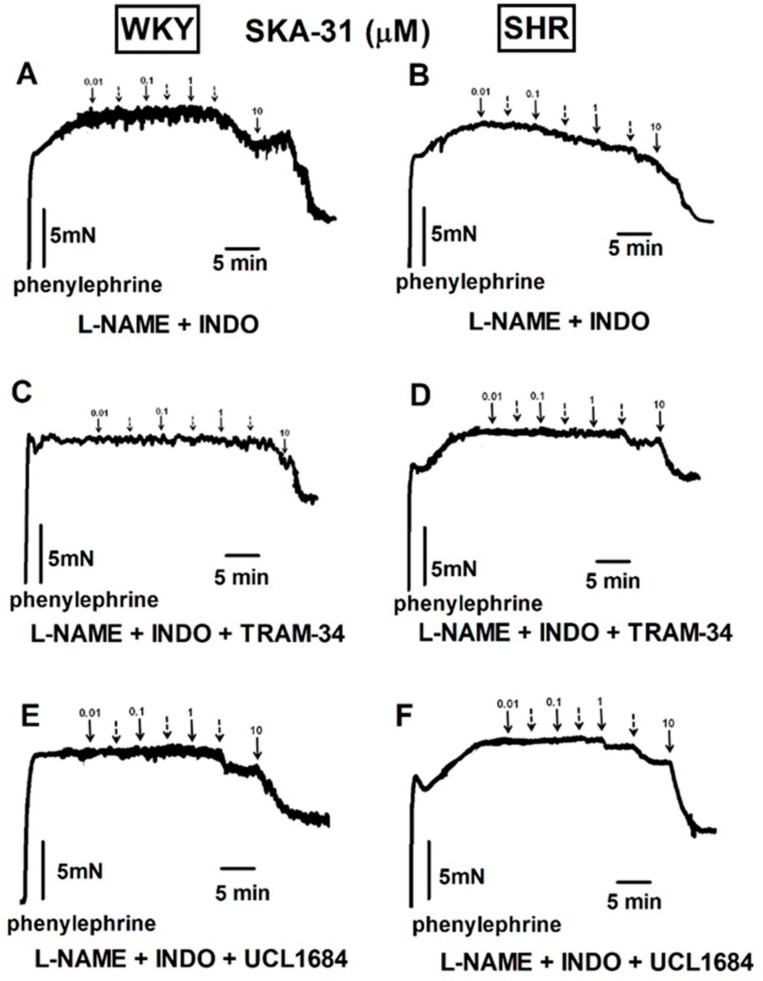
Traces from representative experiments showing the influence of indomethacin (INDO, 10 μM) and NAME -*l*-arginine methyl ester (*l*-NAME, 100 μM) given together (**A**,**B**), TRAM-34 (10 μM; **C**,**D**) or UCL1684 (0.1 μM; **E**,**F**) given in the presence of *l*-NAME and INDO (100 μM and 10 μM, respectively) on the vasorelaxant effect of SKA-31 in the small mesenteric arteries from normotensive WKY (**A**,**C**,**E**) and spontaneously hypertensive (SHR) rats (**B**,**D**,**F**). Arrows show the moment of application of the particular concentrations of SKA-31. Broken arrows represent the following concentrations (from the left): 0.03, 0.3, 3 µM, respectively.

Incubation with *l*-NAME (100 µM) or the combination of *l*-NAME (100 µM) and INDO (10 µM) (EDH) shifted the CRCs for SKA-31 about 15- to 16-fold in WKY rats and 3- to 3.2-fold in SHR but decreased the maximal relaxation effect only in WKY rats. In both groups, the EDH-type relaxation was comparable in normotensive and hypertensive groups (Figure 4A,B, for the pEC_25_ and R_max_ see values in Table 2, the original traces see in Figure 5A,B).

In order to correlate the results of functional analyses with accompanying changes at the molecular level, mRNA expression of endothelial nitric oxide synthase (*eNOS*) and prostaglandin I_2_ synthase (*PGIS*) was evaluated. A RT-qPCR method was used to compare the expression of the abovementioned genes in SHR and WKY rats. Accordingly, the expression of *eNOS* was significantly down-regulated in SHR compared to WKY rats, while there were no statistically relevant changes in *PGIS* expression (Figure 4C).

### 2.4. Influence of K_Ca_, K_IR_2.1, and Na^+^/K^+^-ATPase Inhibitors on EDH-Type Relaxation Induced by SKA-31

All subsequent experiments were carried out in the presence of *l*-NAME (100 µM) and indomethacin (10 µM).

**Figure 6:**
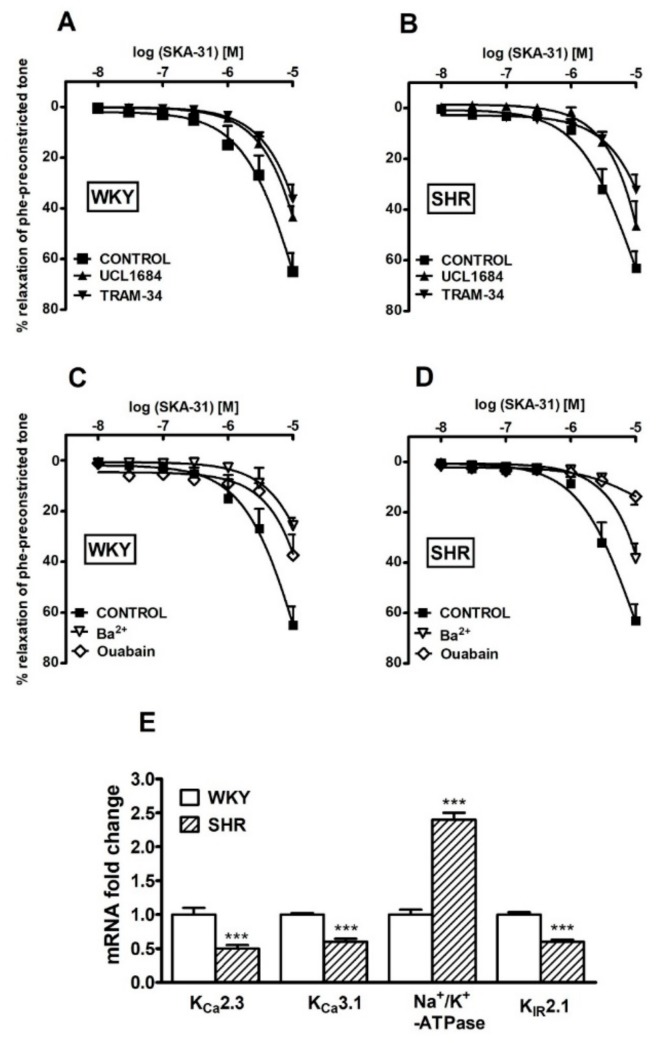
Influence of UCL1684 (0.1 μM), TRAM-34 (10 μM), Ba^2+^ (30 μM) and ouabain (100 μM) on the vasorelaxant effect of SKA-31 in the presence of *l*-NAME and indomethacin (INDO) (100 μM and 10 μM, respectively; CONTROL) in small mesenteric arteries from normotensive Wistar Kyoto (WKY) (**A**,**C**) and spontaneously hypertensive (SHR) rats (**B**,**D**). RT-qPCR analysis of *K_Ca_2.3*, *K_Ca_3.1*, *Na^+^/K^+^-ATP-ase*, and *K_ir_2.1* in vessels isolated from SHR and WKY rats. Results shown as relative fold change in mRNA expression in SHR in comparison to WKY controls, where expression level was set as 1.0 (**E**). Results are expressed as a percentage of relaxation of the isometric contraction induced by phenylephrine (Phe). Mean ± SEM of *n* = 5–14 animals for each curve. In a few cases, the SEM is smaller than or equal to the size of symbols. *** *p* < 0.001 compared to WKY. See Table 2 for statistical analyses.

Inhibitors of *K_Ca_2.3* (UCL1684, 0.1 µM) and *K_Ca_3.1* (TRAM-34, 10 µM) attenuated EDH-type relaxation in the sMAs of WKY by about 30% and 45%, respectively, and in SHR by about 25% and 50%, respectively (Figure 6A,B, for the pEC_25_ and R_max_ see values in Table 2, the original traces see in Figure 5C–F). The expression levels of *K_Ca_2.3* and *K_Ca_3.1* channel mRNA were decreased in SHR in comparison to WKY rats (Figure 6E).

Ba^2+^ (inhibitor of K_IR_, 30 µM) and ouabain (inhibitor of Na^+^/K^+^-ATPase, 100 µM) decreased the potency of SKA-31 in the sMAs in WKY and SHR groups. The inhibitors reduced the potency and the maximal vasodilatory effect of SKA-31 in sMAs by about 60% and 40%, respectively, in WKY rats and 40% and 80%, respectively, in SHR in comparison to EDH groups. (Figure 6C,D, for the pE_25_ and R_max_ see values in Table 2).

At the molecular level, the mRNA expression of *K_IR_2.1* was down-regulated, while *Na^+^/K^+^-ATPase* was upregulated in SHR compared to WKY rats (Figure 6E).

### 2.5. Vasodilatory Effects of SKA-31, NS309, and Acetylcholine in sMAs

**Figure 7:**
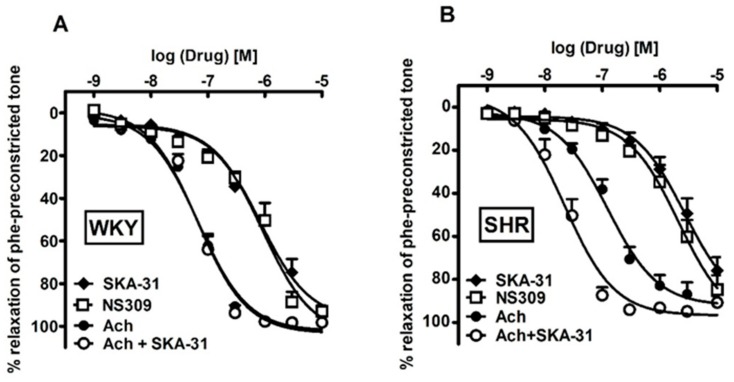
Vasorelaxant effects of SKA-31, NS309, Ach and the interaction of SKA-31 with Ach in small mesenteric arteries from normotensive Wistar Kyoto (WKY) (**A**) and spontaneously hypertensive (SHR) rats (**B**). Results are expressed as a percentage of relaxation of the isometric contraction induced by phenylephrine (Phe). Mean ± SEM of *n* = 5–12 animals for each curve. In a few cases, the SEM is smaller than or equal to the size of symbols.

SKA-31 (0.01–10 µM), NS309 (0.001–10 µM) and Ach (0.001–10 µM) produced a concentration-dependent, nearly complete relaxation in sMAs preconstricted with phenylephrine in WKY (92.8 ± 2.3, 92.9 ± 2.9, 97.2 ± 1.0, respectively) and in SHR (75.8 ± 6.1, 84.7 ± 6.7, 90.5 ± 3.9, respectively) (Figure 7A,B). The rank order of potencies (pEC_50_) of ligands in the relaxation experiments in WKY versus SHR was: Ach (7.2 ± 0.08, *n* = 11 vs. 6.9 ± 0.05, *n* = 11; *p* < 0.01) > SKA-31 (6.0 ± 0.1, *n* = 12 vs. 5.5 ± 0.10, *n* = 13; *p* < 0.01) ≥ NS309 (6.0 ± 0.1, *n* = 5 vs. 5.6 ± 0.07, *n* = 7; *p* < 0.05). Incubation with SKA-31 (0.1 µM) enhanced the potency of Ach-mediated relaxation only in the SHR group and did not affect the maximum relaxation response (Figure 7A,B, pEC_50_ for Ach 6.9 ± 0.05, *n* = 11; SKA-31 + Ach 7.6 ± 0.10, *n* = 10; *p* < 0.01).

## 3. Discussion

Dysfunction of the endothelium is often associated with elevated BP [21] and reduced EDH-mediated relaxation that may be associated with defects in the *K_Ca_3.1*/*K_Ca_2.3*-EDH–dilator system [1], for which SHR is a model [22]. Functional evidence for targeting pathway of impaired *K_Ca_3.1*/*K_Ca_2.3*-EDH–dilator system would raise the possibility to improve vascular function [3] and could serve the way for new treatment for hypertension and related cardiovascular diseases [9]. Therefore, we investigated the *K_Ca_3.1*/*K_Ca_2.x* channel activator SKA-31-induced systemic hemodynamic effects and the putative endothelium-dependent relaxation, including EDH-*K_Ca_2.3*/*K_Ca_3.1* type, in isolated endothelium-intact sMAs in a rat genetic model of primary hypertension in order to check if it could be relevant for antihypertensive therapy/ beneficial in cardiovascular system in hypertension.

### 3.1. Vascular Changes Related to Hypertension

To examine the effects of SKA-31, we conducted experiments using the SHR model, which shares features of primary hypertension observed in various animal species and in humans such as elevated BP, thickening of the media of sMAs, and impaired endothelial function revealed by higher levels of vWF [23,24].

### 3.2. Influence of SKA-31 on BP and HR

In this study we demonstrated that direct i.v. administration of SKA-31 dose-dependently (1, 3, 10 mg/kg) produced transient, rapid decreases in BP in both normotensive and hypertensive rats, and this decrease was higher in SHR than in WKY rats at two lower doses of the compound. Only the highest dose of SKA-31 used (10 mg/kg) evoked profound and short-term decreases in HR amounting to roughly 50% and 40% of basal HR values in SHR and WKY rats. It is highly possible that reduction in BP might be due to decrease in peripheral vascular resistance, and in addition at 10 mg/kg of SKA-31 could be a consequence of decrease in HR and cardiac output. Clearly, the anesthesia has an impact on systemic BP in these animals. The recorded systolic and diastolic BP under urethane anesthesia are much lower than expected, however in general agreement with those previously measured in age-matched male WKY and in SHR [25,26,27]. Moreover, urethane anesthesia is preferred over pentobarbitone since urethane, unlike pentobarbitone, does not affect reflex responses [28], that could be responsible for short-lasting bradycardia and hypotensive effect, observed in this study.

Thus far, no other study appears to report drop in HR by 10 mg/kg of SKA-31 in anesthetized animal models. The reported bradycardic responses in conscious animals [13,16] tend to occur at much higher doses and likely reflect non-vascular, central actions of SKA-31, which was probably due to a centrally mediated decrease in sympathetic drive through activation of neuronal *K_Ca_2.x* channels, as well as possible direct effects on *K_Ca_2.x* channels in cardiac pacemaker tissue [16,29]. Moreover, *K_Ca_2.x* channel has a role in atrial, atrioventricular nodal, and ventricular conduction, and their increased expression enhances the risk of sudden cardiac death due to bradyarrhythmias [30,31]. Regarding the fact, that dose estimation always requires careful consideration of the difference in pharmacokinetics and pharmacodynamics among species [32] and that hypotensive effect of SKA-31 is dose-dependent, it might be possible that bradycardic effects observed in WKYs and SHRs treated with 10 mg/kg SKA-31 is rather a consequence of reflex responses and/or disturbances of cardiac K_Ca_ instead of urethane anesthesia itself. One should keep in mind, that despite urethane is suitable for maintaining anesthesia during electrophysiological recording and has minimal effect on the cardiovascular system, it could also exert marked effects on the ion channels and alter several neurotransmitter systems in high concentration [33,34]. Therefore, further research will be necessary to get to know, to what extent anesthesia impacts the effectiveness or actions of acutely administered SKA-31, and more in-depth examinations will be required to characterize the precise mechanisms of bradycardia associated with i.v. administration of SKA-31.

### 3.3. Influence of SKA-31 on Vasodilatory Effects in sMAs

In rat endothelium-intact sMAs we demonstrated that SKA-31 caused a concentration-dependent relaxation in hypertensive and normotensive rats. The potencies (SHR, 5.5 and WKY, 6.0) and the efficacies (SHR, 75% and WKY, 92%) for SKA-31 were diminished in SHR in comparison to WKY. Surprisingly, contradictory effects were observed in vivo experiments where low doses of SKA-31 (i.e., 1 and 3 mg/kg) were administered to anesthetized rats. Here, SKA-31 induced greater hypotensive effects in SHRs vs. WKYs. This results, although inconsistent with the in vitro analyses, might be explained by higher basal values of cardiovascular parameters in SHRs in comparison to WKYs. We also hypothesize, that other factors such as drug pharmacokinetics, dysfunction within the vasculature and heart, or hormonal/autonomic regulation of these systems in SHRs may lead to homeostatic overcompensation, and subsequent exaggeration of the BP response.

In our study, SKA-31 in normotensive rat sMAs was about 10 times less potent than in cannulated and pressurized small mesenteric arteries [20] and comparably potent to myogenically contracted rat cremaster and cerebral arteries (1.8–2.0 μM) [19]. Vasodilation elicited by other activators of endothelial *K_Ca_2.3* and *K_Ca_3.1* channels, NS309 and Ach, was similar or stronger than SKA-31, however in both cases the response was attenuated in the SHR. Presumably attenuated vasodilatory effect in SHR is not dependent on enhanced degree of contraction tone induced by phenylephrine at 10 µM, since it was comparable to that observed in WKY. Our assumptions were also confirmed by a set of previous research [35]. The small molecule activators of endothelial K_Ca_ channels are effective vasodilators of arteries in several vascular beds (cerebral, coronary, mesenteric) in rats and mice [18,19,22] with maintained, but impaired, responses in hypertension [36,37,38].

Endothelium is a key site of action for SKA-31 because its physical disruption weakens the relaxation response in WKY rats and to a greater extent, in SHR. These results agree with other observations describing the endothelial dependence of SKA-31-mediated relaxation of third-order mesenteric arteries from Sprague-Dawley rats [20] or rat cremaster and cerebral arteries [19]. Of note, SKA-31 had no sizeable impact on endothelium-independent vasodilation to sodium nitroprusside, confirming that there is no interference with endothelium-independent smooth muscle cell functions in mice carotid arteries [37].

Little is known about the effects of SKA-31 as modulator of the EDH-mediated response, thus we decided to study its vascular effects under COX or NOS inhibition. The SKA-31 efficacy was reduced in sMAs isolated from WKY and SHR. Indomethacin or *l*-NAME right-shifted the SKA-31 CRCs in the sMAs by about 5- or 15-fold in WKY and 2.5- or 3-fold in SHR groups, respectively. This suggests that NO, and to a lesser extent PGI_2_, is involved in the vasorelaxant action of SKA-31, especially in normotensive animals. In addition, the reduced SKA-31-mediated vasorelaxation could correlate with lower expression of eNOS in SHR than in WKY rats and with no significant difference in expression of PGIS in both groups. The inhibition of SKA-31-evoked dilation under COX and NOS inhibition was observed in murine isolated perfused kidneys [36]. The K_Ca_ channel activator NS309 mediated an increase in coronary blood flow in dogs [39] and additionally, *K_Ca_2.3* and *K_Ca_3.1* channels were essential for NO-mediated vasorelaxation [40,41]. Using arterial pressure myograph, other investigators have demonstrated SKA-31-evoked inhibition of myogenic tone in rat cremaster, middle cerebral arteries [19], and mesenteric arteries [20] in normotensive animals while hypertensive Cx40- or *K_Ca_3.1*-deficient mice were not affected by the eNOS inhibitor *l*-NAME. Differences in NO involvement in K_Ca_ channel activator-mediated effects might result from experimental procedures and/or nature of the vasoconstrictor stimulus used [42,43]. Thus, it has been recently suggested, that a link between K_Ca_ channel-mediated hyperpolarization and NO production in regulation of smooth muscle cell contractility exists [42,44]. However, it warrants further investigation.

### 3.4. Involvement of K_Ca_2.3 and K_Ca_3.1 Channels in EDH-Type Relaxation Induced by SKA-31

The potency of EDH-related relaxation evoked by SKA-31 was comparable in normotensive and hypertensive rats. Similarly, EDH-type relaxation was the same in murine isolated perfused kidneys of WKY and stroke-prone spontaneously hypertensive rats (SHRSP) [36]. In hypertension, under reduced bioavailability of NO, and when vasorelaxation is impaired, EDH-type dilatation is an important compensatory dilator system [1]. This finding is in line with research using the experimental model of acquired angiotensin II-induced, deoxycorticosterone acetate-salt (DOCA-salt) hypertension in SHR and SHRSP [11,38,45,46] respectively.

The potency and maximal EDH-type vasodilatory action of SKA-31 were attenuated by UCL1684, the selective inhibitor of *K_Ca_2.3*, and TRAM-34, the selective inhibitor of *K_Ca_3.1*, respectively, in both rat strains. As SKA-31 has a 10-fold higher affinity to *K_Ca_3.1* vs. *K_Ca_2.x* channels, which favors activation of *K_Ca_3.1* at SKA-31 concentrations <3 uM. Similar observations have also been reported in murine cremaster muscle arterioles [16] and carotid arteries [13] where SKA-31-induced EDH was wholly dependent on *K_Ca_3.1* activation. In agonist-induced EDH responses and in vasodilation of resistance arteries induced by Ach, *K_Ca_3.1* was the predominant K_Ca_ channel reportedly involved [7,11,12,47]. However, our results are in opposition to the findings that *K_Ca_2.3* plays the main role in the EDH-mediated response in superior mesenteric arteries in SHRSP [38] and in SMAs of angiotensin II-induced hypertension and in SHR [45,48].

Real-time qPCR analysis detected decreased genes expression of *K_Ca_2.3* and *K_Ca_3.1* in SHR compare to WKY rats. Comparable results were obtained on fourth order mesenteric arteries in angiotensin II-induced hypertension and in rat carotid arteries in nephrectomized rats [49]. In contrast to our present findings, Seki et al. [38] reported only significantly decreased peptides of *K_Ca_2.3* and no changes in *K_Ca_3.1* in superior mesenteric arteries in SHRSP. However, studies conducted on second-order mesenteric arteries in SHRSP detected increased expression of *K_Ca_3.1* and decreased expression of *K_Ca_2.3* [50]. The reason for these differences indicates that there may be regional heterogeneity in *K_Ca_2.3* and *K_Ca_3.1* function and gene expression in rat mesenteric beds. It may also be a result of the age of the animals and the vessel on which the experiments were carried out.

Despite reduced mRNA expression levels of *K_Ca_2.3* and *K_Ca_3.1* channels in the sMAs of SHR, SKA-31 maintains endothelial-dependent vasorelaxation at a comparable level to normotensive rats.

### 3.5. Downstream Signaling Pathway in EDH-Type Relaxation Induced by SKA-31

We confirmed that SKA-31 evoked endothelium-dependent vasorelaxation in sMAs from SHR and WKY rats, likely via endothelial hyperpolarization that spreads to adjacent smooth muscle via myo-endothelial gap junctions and further involved the activation of smooth muscle K_IR_ channels and Na^+^/K^+^-ATPase [51].

Activation of Na^+^/K^+^-ATPase and K_IR_ channels contribute to the relaxation of mesenteric arterial smooth muscle cells under EDH conditions. They appear to act as downstream signaling pathways of *K_Ca_3.1* and *K_Ca_2.3*, respectively, to facilitate hyperpolarization of the vascular smooth muscle cells [2]. The functional results obtained correlate with the observed reduction in expression of K_IR_2.1 channels and higher expression of Na^+^/K^+^-ATPases in animals with hypertension. This fact was confirmed by studies conducted by Weston et al. [48], who showed that in the second and third branches of the mesenteric arteries in rats with primary hypertension, the abundance of K_IR_2.1 channels was decreased at the protein level. Based on functional studies, we speculate that the protein expression of Na^+^/K^+^-ATPases increases in SHR, therefore the use of ouabain more effectively inhibits the relaxation of sMAs compared to the control group of animals. The observed effect may also result from the higher selectivity of SKA-31 for *K_Ca_3.1* channels because these interact mainly with Na^+^/K^+^-ATPases. Ouabain inhibits the downstream pathway of the EDH-response preferred by SKA-31.

### 3.6. Influence of SKA-31 on Potentiation of Ach-Induced EDH in sMAs

Pre-incubation with SKA-31 (0.1 µM) enhanced the effect of Ach-mediated vasodilation only in sMAs from SHR. In related studies, SKA-31 has been reported to have potentiating effects on Ach-induced EDH-type vasodilation in pressurized sMAs of WKY and SHR [17], murine carotid arteries, dog first-order mesenteric and rat middle cerebral arteries [13,14,19], and in carotid artery hypertensive *K_Ca_3.1*^−/−^ mice [37]. We hypothesized, that in the presence of a Ca^2+^ mobilizing receptor agonists, such as ACh, K_Ca_ channel activator can certainly augment such actions by boosting or “priming” K_Ca_ channel activities, which was previously shown in isolated endothelial cells and intact vascular tissue [38,52]. In our study, however, this phenomenon occurs only in sMAs of SHR, which theoretically could be the result of the involvement of silent information regulator T1 (sirtuin-1, SIRT1) and AMP-activated protein kinase (AMPK) pathway, that was suggested to restore impaired EDH-mediated relaxation in mesenteric artery at the early stage of hypertension (12 weeks old animals) but not in age-matched WKY [22] or normotensive Sprague-Dawley rats [53]. The activation of AMPK in the regulation of BP and vascular tone has been suggested to be partly dependent on the activation of *K_Ca_3.1* channels and Na^+^/K^+^-ATPase [1]. This is in agreement with higher affinity of SKA-31 to *K_Ca_3.1* vs. *K_Ca_2.x* channels and observed in this study overexpression of Na^+^/K^+^-ATP-ases, downstream pathway of *K_Ca_3.1* channels. The second hypothetical mechanism of enhanced Ach-mediated vasodilatation could be the activation of profound smooth muscle -adrenergic receptor perivascular sympathetic nerves typical for hypertension, which in turns may further overstimulate endothelial K_Ca_ (*K_Ca_3.1*) and increase Ca^2+^ concentrations transmitted from smooth muscle through myoendothelial gap junctions. That is, with SKA-31 stimulated *K_Ca_2.3* and/or *K_Ca_3.1* channel-mediated hyperpolarizing K^+^ currents and NO production acting as a feedback mechanism, the result is dampened contraction in vascular smooth muscle [42,43,54]. Physiologically, the endothelium-dependent feedback could limit increased vascular tone in sustained sympathetic nerve activity in hypertension, including SHR.

### 3.7. Limitations

It is important to mention the following limitations of our study.

In accordance with previous indications that SKA-31 might modulate BP [12], we report the novel finding that single injections of SKA-31 at 10 mg/kg generate an acute reduction in BP and HR enhanced in SHR, resembling that observed in TRPV1-mediated Bezold-Jarisch reflex (e.g., [27]). This assumption might be possible, since TRPV1 receptors, are among other transient receptor potential channels, suggested to interact with K_Ca_ and EDH [43] and are highly implicated in the pathogenesis of hypertension and related cardiovascular diseases [55]. However, it is impossible to precisely determine the character of hemodynamic response of SKA-31, whether it is reflex or nor reflex response, thus more experiments i.e., in pithed and/or vagotomized rats are needed. Furthermore, observed in-vivo hemodynamic responses can be affected by the anesthetic regime utilized during experimental protocols and should be interpreted in this context and repeated in conscious animals.

In order to minimize the abovementioned biases, we decided to use the selective inhibitors (according to [50]). However, TRAM-34 at concentration used in this study 10 µM has been demonstrated to directly block Kv1.3, Kv1.4, Kv7.2 + Kv7.3 and Nav1.4 channels, including significant stimulation of K_Ca_2 channels [56]. The precise mechanisms of SKA-31 mediated vascular effects, including enhancement of Ach-evoked vasorelaxation warrants further investigation and application of more selective antagonists combined with comprehensive molecular analysis at genetic, transcriptomic, and proteomic level. In addition, quantitative RT-PCR results need to be verified by Western blotting.

## 4. Materials and methods

### 4.1. Animals

Male 10–12 weeks old SHR and Wistar-Kyoto (WKY) rats that weighed 280–310 g were purchased from the Center for Experimental Medicine of the Medical University of Białystok. All surgical procedures and experimental protocols were carried out in accordance with the European Directive (2010/63/EU) and Polish legislation and were approved by the local Animal Ethics Committee in Olsztyn (Poland, project code: 81/2017, approved 28 November, 2017). Animal studies are reported in compliance with the ARRIVE guidelines [57]. The study was performed in compliance with the principles of replacement, refinement or reduction (the 3Rs). Animals were housed at constant humidity (60 ± 5%) and temperature (22 ± 1 °C) and were kept under a 12:12 h light–dark cycle. They were maintained on standard pelleted rat chow and tap water ad libitum unless otherwise noted.

### 4.2. Measurements of Blood Pressure (BP)

Systolic blood pressure (SBP) was measured in conscious animals by a non-invasive tail-cuff method (Hugo Sachs Elektronik-Harvard Apparatus, March–Hugstetten, Germany). Measurements are approximations and serve to illustrate a relative difference between SHR and WKY rats, but not precise absolute values for SBP.

### 4.3. In Vivo Effects of SKA-31 in SHR and WKY Rats

Experiments were performed on urethane (14 mM/kg, i.p.) anaesthetized SHR and WKY rats. Following anesthesia, the animals were tracheotomized. The right carotid artery was separated from the vagus nerve and cannulated to measure the SBP and diastolic blood pressure (DBP) via a pressure transducer (Isotec, Hugo Sachs Elektronik, March–Hugstetten, Germany), mean arterial pressure (MAP) was calculated with a standard formula: MAP = DBP + 1/3(SBP – DBP). The heart rate (HR) was recorded using an electrocardiogram. The left femoral vein was cannulated for the intravenous (i.v.) administration of drugs [58]. Each animal was injected with SKA-31 in increasing doses (1, 3, 10 mg/kg) or respective vehicles at a volume of 0.5 mL/kg (1, 3 mg/kg) or 1 mL/kg (10 mg/kg). Subsequent injections were given after returning the cardiovascular parameters to basal values.

### 4.4. Vessel Preparation

Male SHR and WKY rats were anaesthetized with pentobarbitone sodium (300 µM/kg i.p.). Two mm long segments of the third-order mesenteric arteries (small mesenteric arteries, sMAs) were prepared mounted in a Mulvany-Halpern-type wire myograph (Multi Wire Myograph System DMT 620 M, Danish Myo Technology, Aarhus, Denmark) as described previously [11].

After the equilibration period, the arterial integrity of the endothelium was assessed by pre-constricting rings submaximally with phenylephrine (Phe, 10 µM) followed by relaxation with acetylcholine (Ach, 10 µM). We considered a relaxation response of at least 90% to Ach to be an endothelium-intact vessel.

When the endothelium was not required, it was removed by rubbing the intima with a horsehair, and successful endothelial denudation was verified by a lack (≤10%) of vasorelaxant response to Ach (10 µM). After washout, experiments were performed. For each preparation only one experimental curve was determined.

### 4.5. Concentration-Response Curves

Cumulative concentration-response curves (CRCs) to the *K_Ca_2.3* and *K_Ca_3.1* channel activators SKA-31 and NS309 (0.01–10 µM) and Ach (0.001–10 µM) were performed in Phe-preconstricted arteries. To examine the mechanisms involved in the vasorelaxant effect of SKA-31, rings were treated for 30 min with the following inhibitors: indomethacin (inhibitor of cyclooxygenase, COX, 10 µM), *N*^ω^-nitro-*L*-arginine methyl ester (*l*-NAME, inhibitor of nitric oxide synthase, NOS, 100 µM), UCL1684 (*K_Ca_2.x*, 0.1 µM), TRAM-34 (*K_Ca_3.1*, 10 µM), and ouabain (Na^+^/K^+^-ATPase inhibitor, 100 µM), Ba^2+^ (K_IR_2.1 inhibitor, 30 µM) [22,50]. In control tissues, the respective vehicles were used instead. All the inhibitors were present during the construction of the CRCs and did not influence the pre-constricted tone. The phenylephrine-induced vasocontractions were compared between WKY and SHR groups in sMAs (for details see Table 2, in the case of Ach, NS309 and combination with Ach+SKA-31 the tension were 10.4 ± 1.2, *n* = 11; 9.9 ± 1.9, *n* = 11 and 9.5 ± 1.2, *n* = 5; 9.4 ± 0.9, *n* = 6, 10.7 ± 1.7, *n* = 10 in WKY and SHR, respectively).

### 4.6. Histopathological Examination

#### 4.6.1. Width of Tunica Media in Mesenteric Arteries

Third-order mesenteric arteries were fixed in 10% buffered formalin and embedded in paraffin in a routine manner. Four µm thick sections were cut using a Leica 2025 (Leica Biosystems Inc., Buffalo Grove, IL, USA) rotating microtome and stained by hematoxylin and eosin (H+E). Histomorphological analysis of mesenteric arteries slides was performed using an Olympus B×41 microscope (Olympus Corporation, Tokyo, Japan). Digital images were processed using NIS Elements BR software (NIS-Elements BR 3.2 64-bit NIS AR 3.0) which was used to calculate the width of the media. The measurement of the thickness of the middle layer of the mesenteric arteries was made at a uniform 200× magnification. Five sections from each specimen were measured [59].

#### 4.6.2. Immunohistochemistry

In the immunohistochemical study, the EnVision method was used according to Tokajuk et al. [60] using antibodies against von Willebrand factor (vWF) (1:2000–2 h incubation at RT, Polyclonal Rabbit Anti-Human (no cat. A 0082); DakoCytomation, Glostrup, Denmark). Antigen retrieval was performed before commencing immunohistochemical staining for vWF using a Target Retrieval Solution (S1699; Dako, Denmark). A negative control was included in which the antibody was replaced by normal rabbit serum (Vector Laboratories, Burlingame, CA, USA) at the respective dilution (no staining), and a positive control was included using rat lung stained for vWF. The obtained results of immunohistochemical staining were evaluated on an Olympus B×41 microscope with an Olympus DP12 camera under 200× magnification. The intensity of the immunohistochemical reaction was measured using a 0 to 256 grey scale level in which completely black pixels were given a value of 256 and white or bright pixels were given a value of 0.

### 4.7. Real-Time qPCR

Small mesenteric arteries were isolated from nine SHR and nine WKY rats. Tissue samples were immediately flash-frozen in liquid nitrogen and stored at −80 °C. Total RNA was isolated from sMAs pooled from three rats per sample. Up to 5 mg of frozen tissue was finely ground with a chilled stainless-steel mortar and pestle. Total RNA was purified using NucleoSpin^®^ RNA XS (Macherey-Nagel, Düren, Germany) with carrier RNA and rDNase treatment according to the manufacturer’s protocol. Spectrophotometric measurements (A260/A280) were done to assess the quantity and quality of the extracted RNA (NanoPhotometer, Implen, Germany).

cDNA synthesis was performed using the High Capacity RNA-to-cDNA Kit (Applied Biosystems, Foster City, CA, USA) following the manufacturer’s instructions. Briefly, 1 μg of purified total RNA was used in a 20 μL reaction mixture containing random octamers, oligo dT-16 primers, dNTPs and MuLV Reverse transcriptase (RT). cDNA (2 μL) served as a template for real-time qPCR reactions. Amplification of the product was performed using SsoAdvanced Universal SYBR Green Supermix (Bio-Rad, Hercules, CA, USA). Sequences of the PCR primers (Table 3) were previously described as: *K_Ca_2.3*, *K_Ca_3.1* [61], endothelial nitric oxide synthase *eNOS* [62], *Na^+^/K^+^-ATPase, K_IR_2.1* [63], and prostacyclin I_2_ synthase *PGIS* [64]. As an internal control, a set of three housekeeping genes was used: *GAPDH*, β-actin [61] and cyclophilin A [65]. Additional evaluation of the primer accuracy was done using Primer-BLAST tool (http://www.ncbi.nlm.nih.gov/tools/primer-blast). The following reaction parameters were applied: initial denaturation at 95 °C for 3 min followed by 40 cycles of 95 °C for 1 min, 60 to 65 °C for 30 s, and 72 °C for 45 s. The CFX Connect Real-Time PCR System (Bio-Rad) was used to perform a real-time qPCR assay. Reactions were run in triplicates and expression was analyzed using the relative quantification method modified by Pfaffl [66].

### 4.8. Drugs

Cremophor EL, dimethyl sulfoxide (DMSO), and urethane were from Sigma-Aldrich (Steinheim, Germany); NaCl was from POCh (Gliwice, Poland). Acetylcholine chloride, (-)- phenylephrine hydrochloride, *l*-NAME, indomethacin, ouabain, and barium chloride (MP Biomedicals, Santa Ana, CA, USA) were dissolved in deionized water except for indomethacin (dissolved in 0.5 M NaHCO_3_). SKA-31 (Abcam, Cambridge, MA, USA) was dissolved immediately before the in vivo experiments in a mixture of DMSO, Cremophor EL and saline (1:2:7) to obtain a concentration of 10 mg/mL and then it was further diluted with saline to obtain final concentrations of 1 and 3 mg/0.5 mL. Stock solutions (10 µM) of UCL1684, NS309 (Sigma-Aldrich, St.Louis, MO, USA), SKA-31 and TRAM-34 (Tocris Bioscience, Bristol, UK) were prepared in DMSO. The final concentrations of these agents were prepared by dilutions with deionized water which adjusted the final concentrations of DMSO to ≤0.1% *v*/*v* for experiments on isolated organs. None of the solvents used affected basal parameters in vivo or in vitro.

### 4.9. Statistical Analysis

All relaxation effects produced by SKA-31, NS309, and Ach, or their solvents were expressed as the percentage relaxation of the tone induced by Phe.

GraphPad Prism 5.0 software (California, CA, USA) was used to plot the mean data as sigmoidal CRCs. The curves were used to determine potency (pEC_50_ or pEC_25_) and the maximal relaxation effect values (*R*_max_). All results are expressed as the mean ± SEM of *n* animals.

Intergroup statistical comparisons were made by analysis of variance (one-way ANOVA) followed by Dunnett’s multiple comparison test. Student’s *t*-test for unpaired data were used as appropriate. Differences were considered significant at *p* < 0.05.

## 5. Conclusions

In conclusion, the present study revealed that SKA-31 is a potent hypotensive agent and an effective activator of *K_Ca_2.x* and *K_Ca_3.1* channels in sMAs. Despite the decreased expression of these channels at the level of mRNA in primary spontaneous hypertension, SKA-31 maintains EDH-responsive vasodilation at a level comparable to normotensive WKY rats. In hypertension, under reduced bioavailability of NO and when vasorelaxation is impaired, EDH-type dilatation could be an important compensatory dilator system [1]. SKA-31 may act synergistically with other vasodilation mechanisms to reduce BP and/or to potentiate a subset of the feedback processes contributing to agonist-stimulated vasodilation. As was suggested previously, *K_Ca_3.1* and *K_Ca_2.3* channel may represent novel and effective therapeutic targets for the treatment of cardiovascular diseases associated with endothelial dysfunction [12,52]. The effects of K_Ca_ channel activator like SKA-31 may prove therapeutically beneficial in patients in the use of short-acting agonists, e.g., with hypertension resistant to conventional treatment, with hypertension that occurs during surgical procedures, in patients with vasoconstrictor insensitivity to NO donors, or with chronic obstructive pulmonary disease where the use of beta-blockers is contraindicated [47].

## Figures and Tables

**Table 1:** Basal diastolic blood pressure (DBP), systolic blood pressure (SBP), calculated mean arterial pressure (MAP) and heart rate (HR) before i.v. injections of increasing doses of SKA-31 or vehicle in urethane-anaesthetized spontaneously hypertensive rats (SHR) and Wistar Kyoto rats (WKY).

Group	*n*	Before SKA-31 (1 mg/kg)/veh				Before SKA-31 (3 mg/kg)/veh				Before SKA-31 (10 mg/kg)/veh			
		DBP	SBP	MAP	HR	DBP	SBP	MAP	HR	DBP	SBP	MAP	HR
WKY+SKA-31	4	40 ± 2	82 ± 4	54 ± 3	258 ± 7	43 ± 2	86 ± 5	57 ± 3	263 ± 11	45 ± 2	95 ± 5	62 ± 3	262 ± 6
WKY+veh	4	40 ± 4	81 ± 6	54 ± 5	251 ± 19	44 ± 3	86 ± 4	58 ± 3	235 ± 10	44 ± 2	87 ± 2	58 ± 2	235 ± 12
SHR+SKA-31	4	52 ± 4	113 ± 7 **	72 ± 4 *	320 ± 27	55 ± 3	120 ± 5 **	77 ± 4 *	347 ± 33	56 ± 4	121 ± 1	78 ± 3 *	371 ± 31 *
SHR+veh	4	52 ± 3	106 ± 3 *	70 ± 2 *	287 ± 21	51 ± 3	109 ± 3 *	70 ± 3	294 ± 26	54 ± 4	115 ± 4	74 ± 4 *	320 ± 23

Mean ± SEM; * *p* < 0.05, ** *p* < 0.01 compared to respective WKY group. *n* represents the number of animals.

**Table 2:** Contractile responses induced by phenylephrine, pEC_25_ and R_max_ values for the vasorelaxant effects of SKA-31 (0.01–10 µM) determined 30 min after administration of particular inhibitors, channel blockers, or endothelium denudation in isolated small mesenteric G3 arteries from normotensive control (WKY) and hypertensive (SHR) rats.

Group	Concentration in (μM)	ENDO	WKY				SHR			
			*n*	Tension (mN)	pEC_25_	R_max_ (%)	*n*	Tension (mN)	pEC_25_	R_max_ (%)
SKA-31	0.01–10	+	12	10.6 ±1.2	6.8 ± 0.10	92.8 ± 2.3	13	10.7 ± 1.1	6.1 ± 0.07 ^&&&^	75.8 ± 6.1 ^&^
SKA-31	0.01–10	−	11	9.6 ± 1.4	5.2 ± 0.06 ***	33.2 ± 2.1 ***	4	9.0 ± 1.3	N.D.	19.6 ± 7.1 ***^,&^
+INDO	10	+	5	9.5 ± 1.6	6.1 ± 0.07 ***	91.4 ± 6.5	6	10.9 ± 1.5	5.7 ± 0.06 *	81.0 ± 3.2
+*l*-NAME	100	+	5	10.2 ± 1.5	5.7 ± 0.10 ***	68.2 ± 5.4 ***	5	11.0 ± 1.0	5.6 ± 0.13 **	68.1 ± 5.6
+*l*-NAME+INDO (EDH)	100 + 10	+	14	10.7 ± 0.8	5.6 ± 0.07 ***	64.9 ± 7.4 ***	15	11.4 ± 1.0	5.6 ± 0.10 **	63.1 ± 6.6
+UCL1684	0.1	+	9	11.6 ± 1.4	5.3 ± 0.06 ^#^	43.4 ± 4.3	8	10.7 ± 1.5	5.3 ± 0.08 ^#^	46.5 ± 4.0
+TRAM-34	10	+	6	9.3 ± 0.8	5.2 ± 0.10 ^##^	36.4 ± 5.8 ^#^	11	9.1 ± 1.6	5.2 ± 0.07 ^##^	32.2 ± 6.1 ^##^
+Ba^2+^	30	+	9	8.9 ± 1.6	5.0 ± 0.20 ^##^	25.7 ± 3.1^###^	10	10.7 ± 1.1	5.1 ± 0.10 ^##^	38.2 ± 3.9 ^###,&^
+OUABAIN	100	+	10	9.4 ± 1.1	5.2 ± 0.06 ^#^	37.3 ± 3.2 ^##^	11	10.6 ± 1.2	N.D.	13.6 ± 3.3 ^###,&&^

Data are expressed as mean ± SEM. *n* represents the number of animals. Results of relaxation are expressed as percentage of the isometric contraction induced by phenylephrine (10 μM). SKA-31 (activator of *K_Ca_2.x* and *K_Ca_3.1* channels), ENDO, endothelium; +, ENDO intact; −, ENDO denuded; Indomethacin (INDO, inhibitor of cyclooxygenase); *l*-NAME (inhibitor of nitric oxide synthase); UCL1684 (inhibitor of *K_Ca_2.x* channels); TRAM-34 (inhibitor of *K_Ca_3.1* channels); Ba2+ (inhibitor of KIR); ouabain (inhibitor of Na+/K+-ATPase). #,& *p* < 0.05; **,##, && *p* < 0.01; *** *p* < 0.001, compared to the * Control (+ENDO), ^#^ *l*-NAME+INDO, & WKY group; as determined by one-way ANOVA followed by Dunnett’s multiple comparison test and Student’s *t*-test for unpaired data. N.D., not determined because the extent of vasorelaxant effect of SKA-31 is very low under this condition.

**Table 3:** RT-qPCR: Primer sequences.

Primer	Sequence	References
*K_Ca_2.3*	Forward: 5′-CGCCTTCAGAATAGAGTT-3′ Reverse: 5′-GAGTGTGCATTGTATTGG-3′	[61]
*K_Ca_3.1*	Forward: 5′-CTGAGATGTTGTGGTTCCT-3′ Reverse: 5′-CAGTGGACAGCGTGATTA-3′	[61]
*eNOS*	Forward: 5′-AGCATGAGGCCTTGGTATTG-3′ Reverse: 5′-CCCGACATTTCCATCAGC-3′	[62]
Na^+^/K^+^-ATPase	Forward: 5′-GAAGCTCATCATCAGGCGACG-3′ Reverse: 5′-CCAGGGTAGAGTTCCGAGCTC-3′	[63]
K_IR_2.1	Forward: 5′-CACGGGGATCTGGATGCTTCTAAA-3′ Reverse: 5′-AGCAATCGGGCACTCGTCTGTAAC-3′	[63]
PGIS	Forward: 5′-TTTTACAGATGACCGCACTCC-3′ Reverse: 5′-GAAATGAGTCAGCAGCAGGAC-3′	[64]
GAPDH	Forward: 5′-TGACTCTACCCACGGCAAGTT-3′ Reverse: 5′-TGATGGGTTTCCCGTTGATGA-3′	[61]
β-actin	Forward: 5′-GGGAAATCGTGCGTGACATT-3′ Reverse: 5′-GCGGCAGTGGCCATCTC-3′	[61]
cyclophilin A	Forward: 5′-TGTCTCTTTTCGCCGCTTGCTG-3′ Reverse: 5′-CACCACCCTGGCACATGAATCC-3′	[65]

## Abbreviations

Ach	acetylcholine
AMPK	AMP-activated protein kinase
CRCs	concentration-response curves
COX	cyclooxygenase
DBP	diastolic blood pressure
DMSO	dimethyl sulfoxide
EDH	endothelium-derived hyperpolarization
HR	heart rate
i.p.	intraperitoneal
i.v.	intravenous
INDO	indomethacin
K_Ca_	calcium-activated potassium channel
*K_Ca_2.x*	small conductance calcium-activated potassium channels
*K_Ca_2.3*	type 3 endothelial small conductance calcium-activated potassium channels
*K_Ca_3.1*	intermediate conductance calcium-activated potassium channel
K_IR_	inward-rectifier potassium ion channel
*l*-NAME	*N*^ω^-nitro-*l*-arginine methyl ester
NOS	nitric oxide synthase
PGIS	prostacyclin I_2_ synthase
Phe	phenylephrine
SBP	systolic blood pressure
SKA-31	(naphtha(1–d)thiazol-2-ylamine)
SIRT1	silent information regulator T1, sirtuin-1
sMAs	small mesenteric arteries
SHR	spontaneously hypertensive rats
WKY	Wistar-Kyoto
SHRSP	stroke-prone SHR
vWF	von Willebrand factor
